# Candidate mechanisms of acquired resistance to first-line osimertinib in EGFR-mutated advanced non-small cell lung cancer

**DOI:** 10.1038/s41467-023-35961-y

**Published:** 2023-02-27

**Authors:** Juliann Chmielecki, Jhanelle E. Gray, Ying Cheng, Yuichiro Ohe, Fumio Imamura, Byoung Chul Cho, Meng-Chih Lin, Margarita Majem, Riyaz Shah, Yuri Rukazenkov, Alexander Todd, Aleksandra Markovets, J. Carl Barrett, Ryan J. Hartmaier, Suresh S. Ramalingam

**Affiliations:** 1 Translational Medicine, Oncology R&D, AstraZeneca, Boston, MA USA; 2 Department of Thoracic Oncology, H. Lee Moffitt Cancer Center & Research Institute, Tampa, FL USA; 3 Jilin Provincial Cancer Hospital, Changchun, China; 4 Department of Thoracic Oncology, National Cancer Center Hospital, Tokyo, Japan; 5 Department of Thoracic Oncology, Osaka International Cancer Institute, Osaka, Japan; 6 Division of Medical Oncology, Department of Internal Medicine, Yonsei Cancer Center, Yonsei University College of Medicine, Seoul, Republic of Korea; 7 Division of Pulmonary and Critical Care Medicine, Kaohsiung Chang Gung Memorial Hospital, Chang Gung University, Kaohsiung, Taiwan; 8 Medical Oncology Department, Hospital de la Santa Creu i Sant Pau, Barcelona, Spain; 9 Kent Oncology Centre, Maidstone Hospital, Maidstone and Tunbridge Wells NHS Trust, Maidstone, UK; 10 Oncology R&D, AstraZeneca, Cambridge, UK; 11 Oncology Biometrics, Oncology R&D, AstraZeneca, Cambridge, UK; 12 Winship Cancer Institute, Emory University, Atlanta, GA USA

**Keywords:** Cancer, Lung cancer, Targeted therapies

## Abstract

Osimertinib, an epidermal growth factor receptor tyrosine kinase inhibitor (EGFR-TKI), potently and selectively inhibits EGFR-TKI-sensitizing and EGFR T790M resistance mutations. In the Phase III FLAURA study (NCT02296125), first-line osimertinib improved outcomes vs comparator EGFR-TKIs in EGFRm advanced non-small cell lung cancer. This analysis identifies acquired resistance mechanisms to first-line osimertinib. Next-generation sequencing assesses circulating-tumor DNA from paired plasma samples (baseline and disease progression/treatment discontinuation) in patients with baseline EGFRm. No EGFR T790M-mediated acquired resistance are observed; most frequent resistance mechanisms are MET amplification (*n* = 17; 16%) and EGFR C797S mutations (*n* = 7; 6%). Future research investigating non-genetic acquired resistance mechanisms is warranted.

## Abstract (web summary)

In the phase III FLAURA study (NCT02296125), the third-generation epidermal growth factor receptor tyrosine kinase inhibitor (EGFR-TKI) osimertinib provided superior progression-free survival versus comparator EGFR-TKIs in patients with NSCLC. Here, by next-generation sequencing of circulating tumor DNA, the authors assess candidate mechanisms of acquired resistance to first-line osimertinib in patients from the FLAURA trial.

## Introduction

Epidermal growth factor receptor tyrosine kinase inhibitors (EGFR-TKIs) are the recommended first-line treatment for advanced non-small cell lung cancer (NSCLC) harboring EGFR-TKI sensitizing mutations (EGFRm)^1^. Despite initial high response rates to first-line EGFR-TKIs, most patients treated with an EGFR-TKI develop resistance. In approximately 50% of patients treated with a first- or second-generation EGFR-TKI, EGFR T790M resistance mutation was detected^2–6^.

Osimertinib is a third-generation, irreversible, oral EGFR-TKI that potently and selectively inhibits both EGFR harboring EGFRm (Exon 19 deletion [Ex19del]/L858R) and EGFR T790M resistance mutations. In clinical trials, osimertinib has shown efficacy in patients with EGFRm and EGFR T790M NSCLC, including patients with central nervous system (CNS) metastases^7–12^. In the Phase III FLAURA study (NCT02296125), osimertinib provided superior progression-free survival (PFS) versus comparator EGFR-TKIs (erlotinib or gefitinib) in patients with previously untreated EGFRm advanced NSCLC (median 18.9 months versus 10.2 months; hazard ratio [HR] 0.46, 95% confidence interval [CI], 0.37 to 0.57; *P* < 0.001)^11^. Final analysis of overall survival (OS) also demonstrated significantly longer OS with osimertinib versus comparator EGFR-TKI (38.6 months versus 31.8 months; HR 0.80, 95.05% CI, 0.64 to 1.00; *P* = 0.046)^12^.

Mechanisms of acquired resistance to osimertinib when used in the second-line setting in patients with EGFR T790M-positive NSCLC after EGFR-TKI treatment have been identified. To date, the most frequently reported resistance mechanisms to second-line osimertinib, including analyses of circulating-tumor DNA (ctDNA) samples from patients in the Phase III AURA3 study are acquired EGFR mutations (e.g., C797S), and amplification of MET and ERBB2 (HER2)^13–15^. However, mechanisms of acquired resistance to osimertinib used in the first-line setting remain to be fully elucidated. A small-scale analysis of genomic mechanisms of acquired resistance in nine patients with previously untreated EGFRm advanced NSCLC who received osimertinib in the Phase I portion of the AURA study showed no cases of acquired EGFR T790M mutation; potential resistance mechanisms identified included other EGFR mutations and amplification of MET and HER2^16^. In another recent, small, retrospective study of paired tissue samples from patients with EGFRm advanced NSCLC analyzed using next-generation sequencing (NGS), it was identified that off-target resistance in the first-line setting was higher versus in the later-line setting, with MET amplification the most common off-target acquired mechanism to first-line osimertinib^17^. However, the majority of resistance mechanisms to first-line treatment are unknown.

Increased understanding of first-line osimertinib resistance mechanisms is essential to inform future therapeutic decisions for patients with EGFRm advanced NSCLC. In this pre-specified analysis, we report the early-onset candidate mechanisms of acquired resistance from plasma samples collected at progression and/or treatment discontinuation in the FLAURA study, from patients for whom there was also a baseline plasma sample with detectable plasma EGFRm. We additionally report the primary mechanisms of resistance to first-line osimertinib detected in baseline tissue samples from patients with and without detectable plasma EGFRm.

## Results

### Patient characteristics and sample disposition

**Fig. 1:**
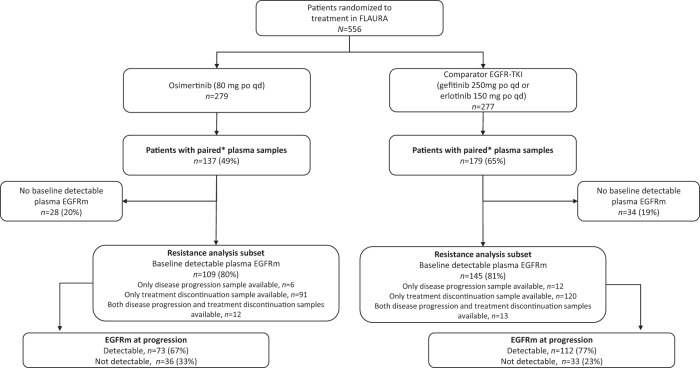
Patient disposition. CONSORT flow diagram of patient disposition and eligibility in the analysis of acquired resistance mutations in the FLAURA trial *Plasma provided at baseline and at disease progression or treatment discontinuation. EGFR epidermal growth factor receptor, p.o. orally, qd once daily, TKI tyrosine kinase inhibitor.

In FLAURA, 279 patients were randomized to osimertinib and 277 patients to comparator EGFR-TKI (gefitinib/erlotinib); 137 (49%) and 179 (65%) patients, respectively, had paired plasma samples analyzed by NGS, i.e. baseline sample and a sample at disease progression and/or treatment discontinuation (Fig. 1). As of June 2017, progression events occurred in 85 (62%) and 147 (82%) patients in the osimertinib and comparator EGFR-TKI arms, respectively.

Among patients with paired plasma samples, 254/316 (80%) had baseline detectable plasma EGFRm (Ex19del/L858R), and were included in the acquired resistance analysis subset: 109/137 (80%) in the osimertinib arm and 145/179 (81%) in the comparator EGFR-TKI arm (Fig. 1). Within this subset, most patients (211/254, 83%) had plasma samples available at treatment discontinuation, compared with a small number of patients with samples only available at disease progression (18/254, 7%) (Fig. 1). There were 25/254 patients (10%) with samples available at both disease progression and treatment discontinuation; only the last collected sample was included in the analysis for these patients (treatment discontinuation, *n* = 13; disease progression, *n* = 12).

There were 224 patients with a discontinuation sample as the last sample collected during the study. Reasons for discontinuation were disease progression (144; 64%), adverse event (26; 12%), patient decision (5; 2%), and one patient (<1%) was reported as ‘other’. No reason was given for 48 patients (21%).

Baseline demographics and clinical characteristics in the acquired resistance analysis subset were broadly similar to the overall FLAURA population and generally balanced between the treatment arms although there was a slightly higher percentage of patients with baseline EGFR Ex19del mutation in the comparator EGFR-TKI arm, compared with the osimertinib arm (Table 1).

**Table 1:** Patient demographics of the overall FLAURA population and the resistance analysis subset^a^

Characteristic	Osimertinib		Comparator EGFR-TKI	
	Overall population (*n* = 279)	Resistance analysis subset (*n* = 109)	Overall population (*n* = 277)	Resistance analysis subset (*n* = 145)
Age: median (range), years	64 (26–85)	62 (26–83)	64 (35–93)	63 (35–93)
Sex: male/female, *n* (%)	101 (36)/178 (64)	43 (39)/66 (61)	105 (38)/172 (62)	48 (33)/97 (67)
Race: Asian/Non-Asian, *n* (%)	174 (62)/105 (38)	71 (65)/37 (34)^b^	173 (62)/104 (38)	92 (63)/53 (37)
WHO performance status: 0/1/2, *n* (%)	112 (40)/167 (60) /0	42 (39)/67 (61)/0	116 (42)/160 (58) /1 (<1)	56 (39)/89 (61)/0
EGFR mutations: Ex19del/L858R/no mutation detected, invalid test, or no or inadequate sample, *n* (%)	158 (57)/97 (35) / 24 (9)	59 (54)/39 (36)/11 (10)	155 (56)/90 (32) / 32 (12)	90 (62)/44 (30)/11 (8)
Histology: adenocarcinoma/other, *n* (%)	275 (99)/4 (1)	108 (99)/1 (1)	272 (98)/5 (2)	142 (98)/3 (2)

*EGFR* epidermal growth factor receptor, *EGFRm* EGFR mutation-positive, *EGFR-TKI* epidermal growth factor receptor tyrosine kinase inhibitors, *Ex19del* Exon 19 deletion, *WHO* World Health Organization.

^a^Subset of patients with detectable baseline plasma EGFRm who progressed or discontinued treatment up to March 2019.

^b^One patient in the resistance analysis subset of the osimertinib arm had missing racial data. In the overall population, five patients (two in the osimertinib arm and three in the comparator EGFR-TKI arm) had large-cell carcinoma; three patients (one in the osimertinib arm and two in the comparator EGFR-TKI arm) had adenosquamous carcinoma; and one patient (in the osimertinib arm) had a carcinoid tumor.

### Acquired resistance mechanisms by treatment arm (plasma ctDNA analysis): Osimertinib arm

**Fig. 2:**
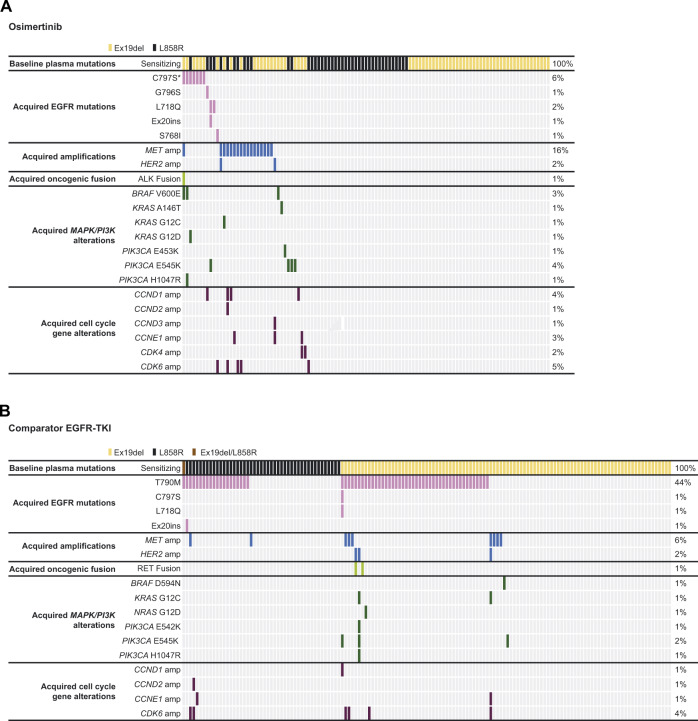
Acquired mutations following treatment with osimertinib and comparator EGFR-TKIs. Tile plots indicating (**A**) acquired mutations in patients treated with osimertinib (*n* = 109) and (**B**) acquired mutations in patients treated with comparator EGFRI-TKIs (*n* = 145) from the FLAURA trial. Source data are provided in the Supplementary Data 1 file. *One patient had co-occurring C797S and C797N. EGFR epidermal growth factor receptor.

In the osimertinib arm acquired resistance analysis subset, 38/109 (35%) patients had a detectable acquired resistance mechanism, 71 (65%) had no detectable candidate mechanism of resistance, and there was no evidence of acquired EGFR T790M. The most common acquired resistance mechanism detected was MET amplification, occurring in 17 patients (16%), followed by mutations in EGFR in 11 patients (10%) (Fig. 2A, Supplementary Table 1). C797S occurred in seven patients (6%), including one patient with co-occurring C797S and C797N; L718Q occurred in two patients (2%; one patient had concurrent L718V), G796S in one patient (1%) and S768I in one patient (1%). Single acquired resistance mechanisms were detected in 23 (21%) patients, including nine (8%) with MET amplification, five (5%) with EGFR mutations, three (3%) with PIK3CA E545K, and one (1%) patient each with CDK6 amplification, CDK4 amplification, CCND1 amplification, KRAS A146T, BRAF V600E, and PIK3CA E453K.

More than one acquired resistance mechanism was detected in 15 (14%) patients (Fig. 2A), meaning 39% of all patients with an acquired resistance mechanism had multiple mechanisms detected. Among patients with MET amplifications, five (5%) co-occurred with cell cycle gene alterations (one patient with multiple amplifications in CCND1, CCND2 and CDK6), one (1%) co-occurred with alterations in BRAF (V600E), ALK fusion and EGFR C797S, one (1%) co-occurred with HER2 amplification, and one (1%) co-occurred with KRAS G12C. Among patients with EGFR mutations, and without co-occurring MET amplification, three (3%) co-occurred with MAPK/PI3K alterations, one (1%) co-occurred with CDK6 amplification and one (1%) with CCND1 amplification. Two (2%) further patients had co-occurring cell cycle gene alterations, CCNE1/CDK4 amplifications and CCNE1/CCND3 amplifications, with the latter also co-occurring with HER2 amplification.

### Acquired resistance mechanisms by treatment arm (plasma ctDNA analysis): comparator EGFR-TKI arm

In the comparator EGFR-TKI arm acquired resistance analysis subset, 71/145 (49%) patients had a detectable acquired resistance mechanism, 74 (51%) had no detectable candidate mechanism of resistance. The most common acquired resistance mechanism detected was EGFR T790M mutation, occurring in 64 (44%) patients, followed by MET amplification in nine patients (6%) and CDK6 amplification in six (4%) patients (Fig. 2B, Supplementary Table 1). Acquired EGFR T790M was found in a similar proportion of patients with baseline plasma Ex19del (44/98 [45%]) versus L858R (19/46 [41%]); one patient (1%) with baseline Ex19del and L858R acquired EGFR T790M. Single acquired resistance mechanisms were detected in 57 (39%) patients, including 51 (35%) with EGFR T790M, four (3%) with MET amplification, and one (1%) patient each with PIK3CA E545K and BRAF D594N.

More than one acquired resistance mechanism was detected in 14 patients (10%), all except one with EGFR T790M (Fig. 2B), and overall, 20% of patients with an acquired resistance mechanism had multiple mechanisms detected. Among seven (5%) patients with cell cycle gene alterations plus EGFR T790M, three (2%) had co-occurring MET amplification, and one (1%) had co-occurring PIK3CA E545K, EGFR C797S, and EGFR L718Q. Among two (1%) patients with HER2 amplification plus EGFR T790M, one (1%) had co-occurring RET fusion, and one (1%) had multiple co-occurring MAPK/PI3K alterations. In one patient without EGFR T790M, co-occurring alterations were detected in MET, HER2, KRAS, CCNE1 and CDK6.

### Duration of treatment by candidate resistance mechanism

**Fig. 3:**
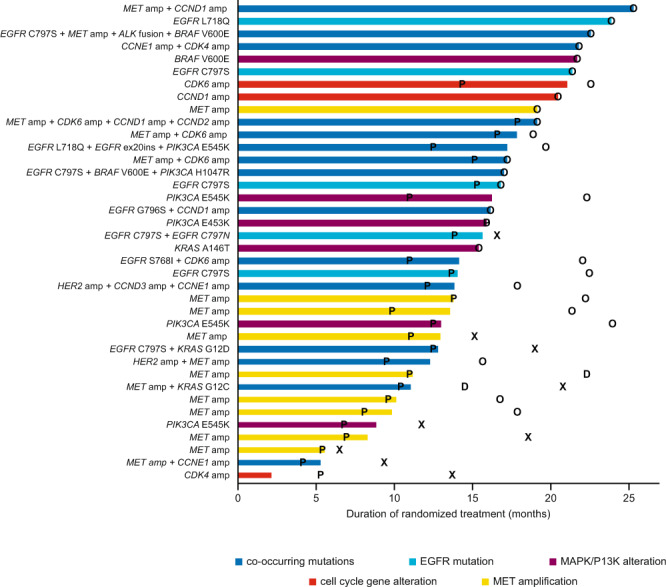
Osimertinib duration of treatment by candidate resistance mechanisms. Swimmer plot indicating duration of treatment with osimertinib (months) by resistance mechanisms (*n* = 109 total, *n* = 38 with detected resistance mutation). Source data are provided in the Supplementary Data 1 file. EGFR epidermal growth factor receptor, X time of death for patients who have died, O date last known alive for patients who have not died, P time of progression, as assessed by investigator, D time of study discontinuation.

Among patients in the resistance analysis subset, there appeared to be no clear association between the type of acquired resistance mechanism and duration of treatment with either osimertinib or comparator EGFR-TKIs (Fig. 3, Supplementary Fig. 1), although in the osimertinib arm, acquired MET amplification occurred in 11 of the 15 patients with the shortest duration of treatment.

### Resistance mechanisms at baseline (tissue genomics)

Across both treatment arms, 147 patients had sufficient tissue volume available and consented for baseline NGS testing, with a pass/qualified result obtained from 104 patients (71%; osimertinib *n* = 46, comparator EGFR-TKI *n* = 58). Reasons for a failed test result included insufficient tumor content (*n* = 24) or insufficient DNA extraction (*n* = 13); six samples were recorded as ‘other’.

In addition to EGFR mutations, the most commonly altered genes included TP53 (62%), EGFR amplification (20%), RB1 (12%), RBM10 (5%), HER2 (3%), MET (3%), SMARCA4 (3%), and RICTOR (3%) (Table 2). TP53 mutations were distributed between missense alterations (28/64; 44%) and loss-of-function mutations (frameshift, truncation, splice site, homozygous deletions; 36/64; 56%).

**Table 2:** Summary of baseline genomic alterations in patients with valid tissue NGS result^a^ and by suboptimal^b^ tumor response to treatment or not

Gene/mutation, *n* (%)	All evaluable (*N* = 104)	Suboptimal^b^ responder (*n* = 23)	Not suboptimal^b^ responder (*n* = 81)
TP53 any known/likely	64 (62)	15 (65)	49 (60)
TP53 frameshift/truncation	26 (25)	2 (9)	24 (30)
TP53 splice	9 (9)	2 (9)	7 (9)
TP53 missense	28 (27)	11 (48)	17 (21)
TP53 homozygous deletion	1 (1)	0	1 (1)
EGFR amplification	21 (20)	7 (30)	14 (17)
RB1 any known/likely	12 (12)	4 (17)	8 (10)
RBM10 truncation/splice	5 (5)	4 (17)	1 (1)
SMARCA4 missense/truncation	3 (3)	3 (13)	0
RICTOR amplification	3 (3)	3 (13)	0
HER2 amplification/missense	3 (3)	3 (13)	0
MET amplification	3 (3)	2 (9)	1 (1)
AKT2 any known/likely	2 (2)	2 (9)	0
CDK6 any known/likely	2 (2)	2 (9)	0
FGF23 any known/likely	2 (2)	2 (9)	0
BRCA2 any known/likely	2 (2)	2 (9)	0
APC any known/likely	6 (6)	3 (13)	3 (4)

*EGFR* epidermal growth factor receptor.

^a^Known/likely refers to alterations either known or likely to have a functional impact on a given protein, as determined by the algorithm described in Carr et al.^49^.

^b^Patients whose tumors had a suboptimal response were defined by either a best overall response of stable disease or progressive disease with a PFS of <6 months, or non-clearance of plasma ctDNA measured by ddPCR at 6 weeks.

Patients whose tumors had a suboptimal response to treatment (*n* = 23 among patients with baseline tissue results) were defined by either a best overall response of stable disease or progressive disease with a PFS of <6 months, or non-clearance of plasma ctDNA measured by ddPCR at 6 weeks. Tumors with suboptimal responses were enriched for TP53 missense alterations and alterations in RBM10, HER2, MET, SMARCA4, or RICTOR compared with tumors not defined as suboptimal response (*n* = 81) (Table 2). Taken together, an alteration in at least one of these potential negative prognostic genes (*n* = 38 tumors in total) was significantly enriched in suboptimal response tumors: 20/23 (87%) vs 18/81 (22%) tumors not defined as suboptimal response; Fisher’s exact test, two-sided *P* < 0.001. EGFR amplification and any TP53 mutation status (either alone or in combination with RB1) were not significantly enriched in tumors with suboptimal responses (Table 2).

## Discussion

This pre-specified analysis from the FLAURA study provides further characterization of the pre-existing and acquired resistance mechanisms to first-line osimertinib. Pre-treatment tissue genomics identified potential baseline alterations associated with suboptimal response to EGFR-TKI therapy, including missense TP53 mutations, RBM10, HER2, MET, SMARCA4, and RICTOR, many of which have previously been identified as negative prognostic factors for patients with cancer^18–23^. Further research to confirm these potential pre-treatment markers of suboptimal response is warranted.

Due to a lack of tissue samples at progression, acquired resistance mechanisms were analyzed using ctDNA. Paired samples were analyzed from patients with detectable baseline plasma EGFRm who experienced disease progression and/or discontinued treatment, revealing candidate mechanisms of acquired resistance in both the osimertinib and comparator EGFR-TKI arms. Consistent with previous studies, EGFR T790M was the most common acquired resistance mechanism in the comparator EGFR-TKI arm, occurring in 44% of patients. Acquisition of EGFR T790M was not observed in the osimertinib arm, supporting the mechanism of action for osimertinib^11,13,14,16,24–27^. This is consistent with preclinical data, indicating that osimertinib may prevent the emergence of EGFR T790M^7,28^. To date, no evidence of acquired EGFR T790M has been observed with first-line osimertinib treatment, and in some cases, resistance to later-line treatment with osimertinib has been associated with loss of EGFR T790M from tumors^15,27^.

Overall, the resistance mechanisms to first-line osimertinib observed in this study appear to be similar to those reported in the second- or later-line settings^12,21,22,26–33^. Our data also demonstrate that there are some shared resistance mechanisms between first-line osimertinib and first- and second-generation EGFR TKIs, with the main exception of EGFR T790M^2,34^. The frequency of MET amplification (16%) was similar to that observed in patients treated with second-line osimertinib in the Phase III AURA3 study (18%)^14,15^, and was the most frequently reported resistance mechanism in both this study and AURA3. Of note, acquisition of MET amplification in this analysis appears more often in osimertinib treated patients compared with first- or second-generation EGFR-TKIs. While the frequency of MET amplification reported here is higher than previously reported with osimertinib in the first-line setting^17^, other second-line osimertinib studies have reported acquired MET amplification in 5–50% of patients with disease progression^26,27,31,33^.

Acquired EGFR C797S, an on-target resistance mechanism that occurs following treatment with irreversible inhibitors, is frequently reported with second- or later-line osimertinib treatment, with some studies reporting frequency rates up to 24%^26,27,31,33^. An analysis of second-line osimertinib in the Phase III AURA3^14,15^ study identified 14% of patients acquired EGFR C797S. In this analysis, acquired C797S mutation was observed in 6% of patients, in line with another study exploring resistance mechanisms to osimertinib in the first-line setting^17,35^. Development of EGFR C797S resistance may be more common in later-line EGFR T790M-positive disease and has been shown to be a later event in the second-line osimertinib setting^17,35^, suggesting that EGFR C797S is a late occurring resistance mechanism. The frequency of acquired PIK3CA, KRAS and BRAF mutations was also generally similar to that reported in second-line osimertinib studies^14,15,27,31,33^.

Apart from EGFR T790M, the acquired resistance mechanisms such as MET amplification and HER2 amplification reported here are also consistent with the acquired resistance mechanisms observed with first- and second-generation EGFR-TKIs^34,36^.

As many patients developed multiple resistance mechanisms, it is difficult to draw any conclusions regarding duration of treatment and the resistance mutations that were acquired. Although patient numbers are small, MET amplification was identified in 11 of the 15 patients with the shortest duration of treatment, suggesting that MET amplification could be enriched in patients with early disease progression.

Importantly, although our understanding of the new resistance landscape with first-line osimertinib is emerging, no suggestions of new and more aggressive resistance mechanisms were detected, although, as discussed below, there were some limitations with the methodology used in detecting all possible resistance mechanisms. This finding, along with the absence of EGFR T790M development, supports the use of osimertinib in the first-line setting. This is reinforced by the overall population data, demonstrating that first-line osimertinib resulted in longer PFS, OS, and time from randomization to second progression on subsequent treatment versus comparator first-line therapy with a similar safety profile^11,12^. Furthermore, osimertinib has demonstrated CNS efficacy in patients with stable CNS metastases and EGFRm advanced NSCLC, with first-line osimertinib reducing the risk of CNS progression compared with first-generation EGFR-TKIs^9,10,37^.

The results suggest a need to identify biomarker-matched treatments to target specific mechanisms of acquired resistance, or to prevent the emergence of these mechanisms. For example, data from the Phase Ib TATTON study (NCT02143466) provide evidence for the potential use of osimertinib in combination with the MET inhibitor, savolitinib, therefore targeting one of the most frequent acquired resistance mechanisms to osimertinib observed in this study: MET amplification. This combination demonstrated promising preliminary anti-tumor activity in patients with MET-amplified advanced NSCLC, who experienced progression on prior first-, second-, or third-generation EGFR-TKIs^38,39^. Those who had progressed on prior first-/second-generation EGFR-TKIs achieved a median duration of response of 7.1 months, with an objective response rate of 52%^39^. SAVANNAH (NCT03778229), a Phase II study assessing the efficacy of osimertinib plus savolitinib in patients with EGFR-mutant, MET-amplified NSCLC who have progressed on osimertinib is investigating this combination further^40^.

When considering the EGFR C797S mutation, first-generation EGFR-TKIs such as gefitinib do not require irreversible binding to C797 to inhibit EGFR so their combination with osimertinib in the first-line setting may be an effective strategy. Preliminary data from preclinical studies have supported this concept with the combination of erlotinib and osimertinib resulting in EGFR signaling inhibition when the T790M and C797S mutations were in the *trans* conformation^41^. Potential new treatment options to address resistance mechanisms to osimertinib will be further explored in the ORCHARD study (NCT03944772), an open-label, multicenter, multi-drug Phase II platform trial in patients with advanced EGFRm NSCLC whose disease has progressed on first-line therapy with osimertinib.

As limited benefit has been demonstrated for immunotherapy alone in the second-line setting for EGFRm advanced NSCLC, other emerging options for patients with acquired resistance include the use of immunotherapy in combination with other therapies^42^. Results from the Phase III IMpower150 study demonstrated that addition of atezolizumab to bevacizumab plus chemotherapy significantly improved PFS and OS in patients with metastatic nonsquamous NSCLC previously treated with at least one EGFR-TKI, regardless of PD-L1 expression and EGFR or ALK genetic alteration status^43^. Alternative approaches following progression on EGFR-TKIs are also being investigated, such as the ongoing Phase Ib/II study investigating the combination of osimertinib and the anti-CD73 monoclonal antibody, oleclumab (NCT03381274)^44^.

There are several limitations with this study. Analysis of plasma ctDNA may underestimate amplification events so the frequency of acquired MET amplification (16%) may be an underestimate and is expected to be higher in tissue^45^. Plasma NGS analysis only focuses on genomic alterations detectable in ctDNA; therefore, non-genetic mechanisms of resistance including histological transformation and protein expression alterations were not evaluated in this analysis; small cell lung cancer transformation could not be pathologically confirmed. In addition, longitudinal monitoring of baseline resistance mutations detectable at progression was not possible due to lack of tissue and plasma matched pairs, NGS panel limitations and no/low ctDNA content in plasma. Other limitations of these analyses include the exploratory nature using a subset of patients defined post-baseline and the exclusion of patients without detectable EGFRm at baseline. Therefore, analyses are descriptive, and there may be additional resistance mechanisms that have not been captured, including any that are late-onset.

In conclusion, multiple mechanisms of resistance to first-line osimertinib were observed, with no single mechanism at high prevalence identified thus far. The most frequent resistance mechanisms were MET amplification and the EGFR C797S mutation, and there was no evidence of EGFR T790M-mediated acquired resistance. These results provide no evidence of resistance mechanisms that may lead to unexpected aggressive disease biology. While liquid biopsy provides valuable information to help monitor and identify emerging resistance mechanisms, the results identify the need for complementary testing with tissue for a complete histological diagnosis. This point is addressed in the ongoing ELIOS study (NCT03239340), where collection of paired tissue biopsies (pre-treatment and at progression) will further investigate the mechanisms of acquired resistance to first-line osimertinib. In addition, the ORCHARD and SAVANNAH studies (NCT03944772, NCT03778229, respectively) are also underway to investigate potential new treatment options to address resistance mechanisms to osimertinib in patients with advanced EGFRm NSCLC whose disease has progressed on first-line therapy with osimertinib.

## Methods

### Standard protocol approvals, registration and patient consents

FLAURA was conducted in accordance with the provisions of the Declaration of Helsinki, Good Clinical Practice guidelines as defined by the International Conference on Harmonisation, applicable regulatory requirements and the AstraZeneca policy on Bioethics and Human Biological Samples. All patients provided written informed consent before screening. This study was funded by the study sponsor (AstraZeneca) and designed by the principal investigators and the sponsor. Data underlying the findings described in this manuscript may be obtained in accordance with AstraZeneca’s data sharing policy described at https://astrazenecagrouptrials.pharmacm.com/ST/Submission/Disclosure. Full study protocol available at: https://astrazenecagrouptrials.pharmacm.com/ST/Submission/View?id=12356.

### Study design and participants

FLAURA was a randomized, double-blind, Phase III study, full details of which have been previously published^12^. Briefly, enrolled patients (*N* = 556) were aged ≥18 years (≥20 years in Japan) with previously untreated, EGFRm locally advanced or metastatic NSCLC with tumors harboring EGFR-TKI sensitizing mutations (Exon 19 deletion [Ex19del] or L858R). Patients were stratified by mutation status (Ex19del/L858R) and race (Asian/non-Asian), and randomized 1:1 to osimertinib 80 mg once daily (qd; *n* = 279) or comparator EGFR-TKI (*n* = 277, gefitinib 250 mg qd or erlotinib 150 mg qd). Patients randomized to comparator EGFR-TKIs were allowed to cross over to osimertinib if they acquired the EGFR T790M resistance mutation and were confirmed to have objective disease progression by a blinded independent central review (or by investigator assessment if disease progression occurred after the primary data cutoff).

The ctDNA and tissue analyses presented here were exploratory, pre-specified, retrospective analyses of a subset of patients. ctDNA analyses were limited to patients who had progressed or discontinued treatment with detectable baseline plasma EGFRm (resistance analysis subset). Patients who did not have detectable plasma EGFRm at baseline were excluded. In addition, 19 patients from China were excluded due to sample export limitations. For the overall FLAURA population, study entry criteria for EGFRm were based on tissue sample analysis.

### Plasma ctDNA and tissue analysis

Serial plasma samples were collected at baseline, 2 weeks, 3 weeks, 6 weeks, 9 weeks, 12 weeks and every 6 weeks thereafter, as well as at disease progression and/or treatment discontinuation. This analysis assessed paired plasma samples collected at baseline and following disease progression and/or treatment discontinuation up to March 2019. Where a sample was available at both progression and at treatment discontinuation, data are reported for the last sample collected, as treatment with osimertinib was allowed following progression. Consequently, samples from patients who remained on treatment post-progression would have been taken after disease progression occurred. Plasma ctDNA samples were analyzed using NGS (Guardant Health, Guardant360 74 gene panel or GuardantOMNI 500 gene panel). All 74 genes on the Guardant360 panel were included in the GuardantOMNI 500 gene panel. The limit of variant allelic fraction detected was 0.04–0.06%. All analyses from each patient (at baseline and following progression and/or treatment discontinuation) were reported only for genes included across both panels used. Genomic alterations were identified using Guardant Health’s proprietary bioinformatics pipeline^46,47^.

Baseline tumor tissue samples were used to analyze co-occurring mutations at baseline that would be associated with suboptimal response to osimertinib. Sufficient tissue for additional genomic analyses from the mandatory tumor tissue biopsy collected during screening was available from 147 patients. Tissue samples were analyzed using the FoundationOne CDx panel^48^.

### Assessments

Disease progression was assessed by the investigator, according to the Response Evaluation Criteria in Solid Tumors (RECIST) version 1.1, every 6 weeks for 18 months, then every 12 weeks until objective progressive disease. After primary data cutoff (June 2017), tumor assessments were carried out in line with clinical practice, and scans were not centrally collected. Acquired mechanisms of resistance were identified in both treatment arms by comparing paired plasma samples at baseline and at disease progression and/or treatment discontinuation in patients with detectable plasma EGFRm at baseline. Primary mechanisms of resistance were identified in both treatment arms from tissue samples collected at baseline in patients with and without detectable plasma EGFRm. Baseline tissue and plasma NGS samples from patients with detectable EGFRm were also compared. Duration of randomized treatment was defined as the time from randomization until end of EGFR-TKI treatment, and was determined for candidate resistance mechanisms in the osimertinib arm and presented as swimmer plots.

### Statistical methods

This pre-specified preliminary analysis was exploratory in nature and, as such, data were summarized using descriptive statistics. Plasma samples at progression or treatment discontinuation included in the paired analysis were collected up until March 2019. Clinical data were analyzed using June 12, 2017 data cutoff; in patients with events occurring after June 12, 2017 were censored in clinical analyses.

### Reporting summary

Further information on research design is available in the Nature Portfolio Reporting Summary linked to this article.

## Supplementary information

Supplementary Information

Description of Additional Supplementary Files

Supplementary Data 1

Reporting Summary

## Data Availability

The de-identified patient data generated in this study are provided in Supplementary Data 1. Specific consent for sequencing data deposition was not obtained from patients. Anonymized patient-level clinical data, aggregated clinical data and/or anonymized clinical study documents underlying the findings described in this manuscript may be obtained in accordance with AstraZeneca’s data sharing policy described at: http://astrazenecagrouptrials.pharmacm.com/ST/Submission/Disclosure. Since at the time of this publication the FLAURA trial is still ongoing, the study data will be accessible at https://vivli.org/ when the trial is completed. In the meantime, requests to access the data from the FLAURA trial described in the current manuscript can be submitted through: https://vivli.org/members/enquiries-about-studies-not-listed-on-the-vivli-platform/. Requested data is available from approval of the request typically for one year. Some patients/countries may need to be excluded based on the informed consent form or country‐level legislation. Use of data must comply with the requirements of Human Genetics Resources Administration of China and patients who have withdrawn consent for data use will be removed from the shared dataset. Patient-level image or genetic data is not available for access. The remaining data are available within the Article and Supplementary Information.
