# Author Correction: Candidate mechanisms of acquired resistance to first-line osimertinib in EGFR-mutated advanced non-small cell lung cancer

**DOI:** 10.1038/s41467-023-38999-0

**Published:** 2023-06-01

**Authors:** Juliann Chmielecki, Jhanelle E. Gray, Ying Cheng, Yuichiro Ohe, Fumio Imamura, Byoung Chul Cho, Meng-Chih Lin, Margarita Majem, Riyaz Shah, Yuri Rukazenkov, Alexander Todd, Aleksandra Markovets, J. Carl Barrett, Ryan J. Hartmaier, Suresh S. Ramalingam

**Affiliations:** 1 Translational Medicine, Oncology R&D, AstraZeneca, Boston, MA USA; 2 Department of Thoracic Oncology, H. Lee Moffitt Cancer Center & Research Institute, Tampa, FL USA; 3 Jilin Provincial Cancer Hospital, Changchun, China; 4 Department of Thoracic Oncology, National Cancer Center Hospital, Tokyo, Japan; 5 Department of Thoracic Oncology, Osaka International Cancer Institute, Osaka, Japan; 6 Division of Medical Oncology, Department of Internal Medicine, Yonsei Cancer Center, Yonsei University College of Medicine, Seoul, Republic of Korea; 7 Division of Pulmonary and Critical Care Medicine, Kaohsiung Chang Gung Memorial Hospital, Chang Gung University, Kaohsiung, Taiwan; 8 Medical Oncology Department, Hospital de la Santa Creu i Sant Pau, Barcelona, Spain; 9 Kent Oncology Centre, Maidstone Hospital, Maidstone and Tunbridge Wells NHS Trust, Maidstone, UK; 10 Oncology R&D, AstraZeneca, Cambridge, UK; 11 Oncology Biometrics, Oncology R&D, AstraZeneca, Cambridge, UK; 12 Winship Cancer Institute, Emory University, Atlanta, GA USA

**Keywords:** Cancer, Lung cancer, Targeted therapies

Correction to: *Nature Communications* 10.1038/s41467-023-35961-y, published online 27 February 2023

The original Article contained errors in the legend of Figure 3 that incorrectly read “*Comparator EGFR-TKI duration of treatment by candidate resistance mechanisms. Swimmer plot indicating duration of treatment with comparator EGFR-TKI (months) by resistance mechanisms (n = 145)*.”. This has been corrected and the legend now reads: “*Osimertinib duration of treatment by candidate resistance mechanisms. Swimmer plot indicating duration of treatment with osimertinib (months) by resistance mechanisms (n = 109 total, n = 38 with detected resistance mutation).”*

Two statements in the Discussion section contained errors and have also been amended.

In the third paragraph, the original sentence *“The frequency of MET amplification (16%) was similar to that observed in patients treated with second-line osimertinib* *in the Phase III AURA3 study (19%)*” should have read “*The frequency of MET amplification (16%) was similar to that observed in patients treated with second-line osimertinib* *in the Phase III AURA3 study (18%)*”.

In the fourth paragraph, the original sentence *“*An analysis of second-line osimertinib in the Phase I AURA^13^ (n=6) and Phase III AURA3^14,15^ studies identified 14% of patients acquired EGFR C797S” should have read “An analysis of second-line osimertinib in the Phase III AURA3^14,15^ study identified 14% of patients acquired EGFR C797S”.

In addition, inadvertently, data from a patient with an acquired BRAF D594N mutation (already included in Figure 2B) were not displayed in Supplementary Figure 1.

The figure and its legend have been updated accordingly in the Supplementary Information file. The original sentence in the Figure legend “*Swimmer plot indicating duration of treatment with comparator EGFR-TKI (months) by resistance mechanisms (n=145 total)”* now reads “*Swimmer plot indicating duration of treatment with comparator EGFR-TKI (months) by resistance mechanisms (n=145 total, n = 71 with detected resistance mutation)*”.

These changes do not affect the scientific conclusions of this work. Errors have been corrected in the PDF and HTML versions of the Article. The HTML has been updated to include a corrected version of the Supplementary Information.

## Supplementary information

Updated Supplementary Information

