# Supplementary material for: Candidate mechanisms of acquired resistance to first-line osimertinib in EGFR-mutated advanced non-small cell lung cancer
Source: Nat Commun. 2023 Feb 27;14:1070. doi: 10.1038/s41467-023-35961-y (PMC9971254; doi:10.1038/s41467-023-35961-y)
Supplement: Supplementary file 1 — Supplementary Information [file 41467_2023_35961_MOESM1_ESM.pdf]

Supplementary Table 1 – Statistical analysis of the frequency of co-occurring mutations detectable in plasma at acquired resistance (FLAURA resistance analysis subset)

Aberration, n	Osimertinib (n=109)		Comparator EGFR-TKI (n-=145)		p-value (Fisher)	p-value (Bonferroni)	Fisher Odds*
	Mutation detected	Mutation not detected	Osimertinib	Comparator EGFR-TKI			
T790M	0	109	64	81	0	0	0
EGFR mutations	11	98	2	143	0.0026	0.026	7.97
MET amplification	17	92	9	136	0.0204	0.204	2.78
HER2 amplification	2	107	3	142	1	1	0.89
RET	0	109	2	143	0.5081	1	0
ALK	1	108	0	145	0.4291	1	Inf
MAPK	6	103	4	141	0.3345	1	2.05
PIK3CA	6	103	3	142	0.1782	1	2.75
Cell cycle	12	97	8	137	0.1565	1	2.11

Aberration, n	Mutation detected		Mutation not detected		p-value (Fisher)	p-value (Bonferroni)	Fisher Odds*
	Osimertinib n=109	Comparator EGFR-TKI n=145	Osimertinib n = 109	Comparator EGFR-TKI n = 145			
T790M	0	64	109	81	0	0	0
EGFR mutations	11	2	98	143	0.0026	0.026	7.97
MET amplification	17	9	92	136	0.0204	0.204	2.78
HER2 amplification	2	3	107	142	1	1	0.89
RET	0	2	109	143	0.5081	1	0
ALK	1	0	108	145	0.4291	1	Inf
MAPK	6	4	103	141	0.3345	1	2.05
PIK3CA	6	3	103	142	0.1782	1	2.75
Cell cycle	12	8	97	137	0.1565	1	2.11

*The conditional Maximum Likelihood Estimate (MLE) rather than the unconditional MLE (the sample odds ratio) is used to calculate odds ratio (fisher.test) in R. Abbreviations: EGFR, epidermal growth factor receptor

Supplementary Fig. 1 | Comparator EGFR-TKI duration of treatment by candidate resistance mechanisms

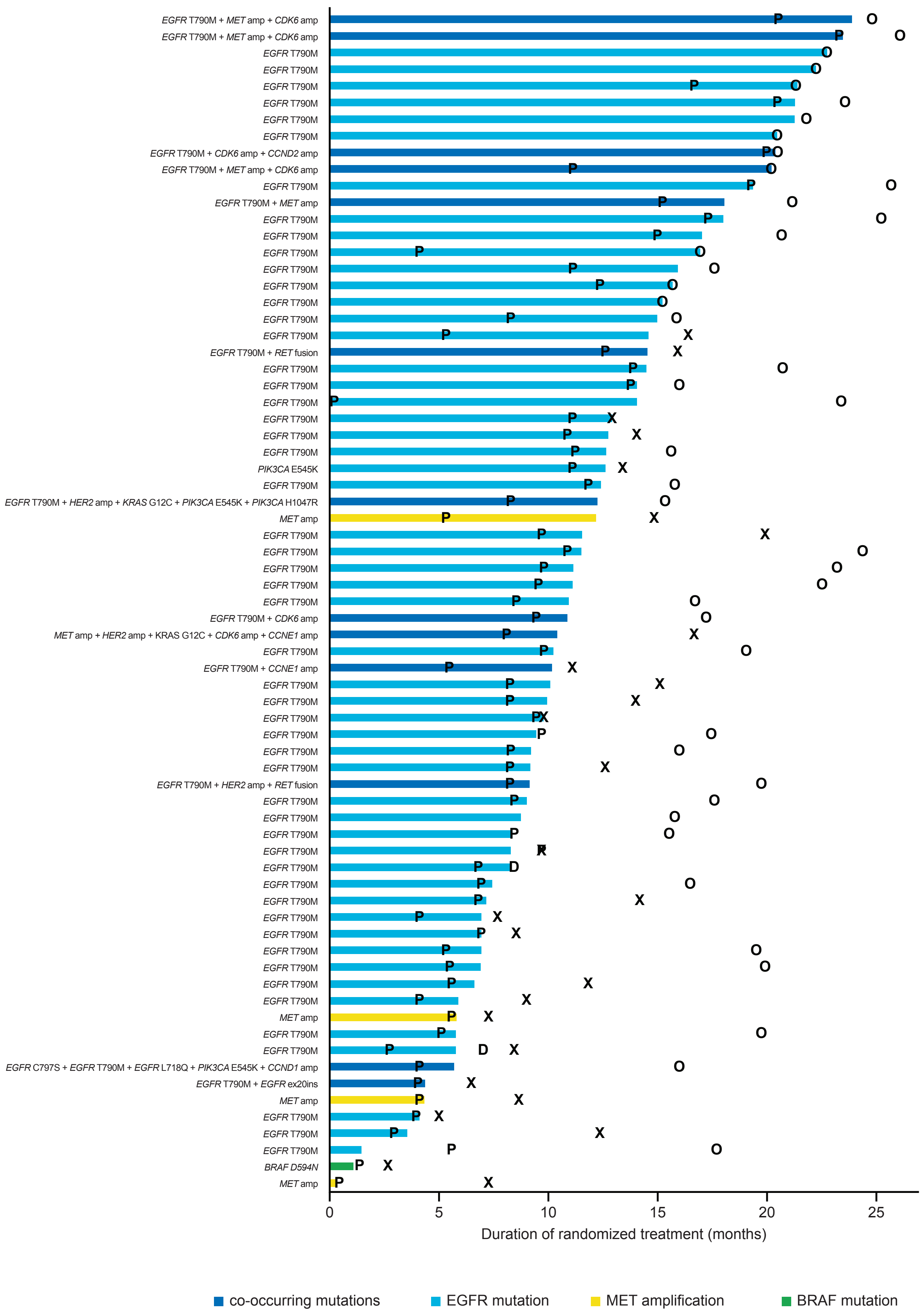

Figure Legend: Swimmer plot indicating duration of treatment with comparator EGFR-TKI (months) by resistance mechanisms (n=145 total, n = 71 with detected resistance mutation). Source data are provided in the Supplementary Data 1 file.

X, time of death for patients who have died; O, date last known alive for patients who have not died; P, time of progression, as assessed by investigator; D, time of study discontinuation.
