# Supplementary material for: Candidate mechanisms of acquired resistance to first-line osimertinib in EGFR-mutated advanced non-small cell lung cancer
Source: Nat Commun. 2023 Feb 27;14:1070. doi: 10.1038/s41467-023-35961-y (PMC9971254; doi:10.1038/s41467-023-35961-y)
Supplement: Supplementary file 4 — Reporting Summary [file 41467_2023_35961_MOESM4_ESM.pdf]

nature portfolio

Corresponding author(s): Dr Jhanelle Gray

Last updated by author(s): Oct 12, 2022

Reporting Summary

Nature Portfolio wishes to improve the reproducibility of the work that we publish. This form provides structure for consistency and transparency in reporting. For further information on Nature Portfolio policies, see our Editorial Policies and the Editorial Policy Checklist.

Statistics

For all statistical analyses, confirm that the following items are present in the figure legend, table legend, main text, or Methods section.

n/a	Confirmed	
☐	☒	The exact sample size (n) for each experimental group/condition, given as a discrete number and unit of measurement
☐	☒	A statement on whether measurements were taken from distinct samples or whether the same sample was measured repeatedly
☐	☒	The statistical test(s) used AND whether they are one- or two-sided<br>*Only common tests should be described solely by name; describe more complex techniques in the Methods section.*
☒	☐	A description of all covariates tested
☒	☐	A description of any assumptions or corrections, such as tests of normality and adjustment for multiple comparisons
☐	☒	A full description of the statistical parameters including central tendency (e.g. means) or other basic estimates (e.g. regression coefficient) AND variation (e.g. standard deviation) or associated estimates of uncertainty (e.g. confidence intervals)
☐	☒	For null hypothesis testing, the test statistic (e.g. F, t, r) with confidence intervals, effect sizes, degrees of freedom and P value noted<br>*Give P values as exact values whenever suitable.*
☒	☐	For Bayesian analysis, information on the choice of priors and Markov chain Monte Carlo settings
☒	☐	For hierarchical and complex designs, identification of the appropriate level for tests and full reporting of outcomes
☒	☐	Estimates of effect sizes (e.g. Cohen's d, Pearson's r), indicating how they were calculated

Our web collection on statistics for biologists contains articles on many of the points above.

Software and code

Policy information about availability of computer code

Data collection	No software used
Data analysis	No software used

For manuscripts utilizing custom algorithms or software that are central to the research but not yet described in published literature, software must be made available to editors and reviewers. We strongly encourage code deposition in a community repository (e.g. GitHub). See the Nature Portfolio guidelines for submitting code & software for further information.

Data

Policy information about availability of data

All manuscripts must include a data availability statement. This statement should provide the following information, where applicable:

- Accession codes, unique identifiers, or web links for publicly available datasets
- A description of any restrictions on data availability
- For clinical datasets or third party data, please ensure that the statement adheres to our policy

The de-identified patient data generated in this study are provided in the Supplementary Information/Source Data file. Data underlying the findings described in this manuscript may be obtained in accordance with AstraZeneca's data sharing policy described at http://astrazenecagrouptrials.pharmacm.com/ST/Submission/Disclosure

Field-specific reporting

Please select the one below that is the best fit for your research. If you are not sure, read the appropriate sections before making your selection.

☒ Life sciences ☐ Behavioural & social sciences ☐ Ecological, evolutionary & environmental sciences

For a reference copy of the document with all sections, see nature.com/documents/nr-reporting-summary-flat.pdf

Life sciences study design

All studies must disclose on these points even when the disclosure is negative.

Sample size	No sample size calculation was performed for this exploratory analysis of resistance mechanisms but the initial sample size for the FLAURA study was determined based on approximately 359 events of progression or death in a total of 530 randomly assigned patients providing at least 90% power to detect a hazard ratio of 0.71 at a two-sided alpha level of 5% (Soria et al. NEJM 2018;387:113-125).
Data exclusions	Only patients who had progressed or discontinued treatment were included in these resistance mechanisms analyses. Patients with a non-detectable plasma EGFR-TKI sensitizing mutation (EGFRm) at baseline were excluded and only patients with paired plasma samples at baseline and at progression and/or treatment discontinuation were included. Patients from China were excluded as plasma samples were unable to be exported for analysis.
Replication	No specific measures were taken to verify the reproducibility of the findings. This manuscript and the accompanying AURA3 resistance sister manuscript demonstrate our attempt to validate the findings in a similar population and there are consistencies in the results (e.g. MET amplification and C797S/fusion mutations). FLAURA findings are being validated in ongoing studies, such as ELIOS and ORCHARD.
Randomization	Patients were stratified according to race (Asian vs non-Asian) and EGFR mutation status (Exon 19 deletion vs L858R) were randomly assigned in a 1:1 ratio to receive osimertinib or comparator EGFR-tyrosine kinase inhibitor (gefitinib/erlotinib).
Blinding	FLAURA was double-blinded but no blinding was used in these exploratory analysis of resistance mechanisms. Blinding of the original study is not especially relevant to this work, but the FLAURA study was blinded. The resistance analysis reported in this manuscript was not blinded because we need to know the actual treatment patients received, in order to interpret resistance mutations - a mechanism of resistance to an unknown therapy is not useful for interpretation.

Reporting for specific materials, systems and methods

We require information from authors about some types of materials, experimental systems and methods used in many studies. Here, indicate whether each material, system or method listed is relevant to your study. If you are not sure if a list item applies to your research, read the appropriate section before selecting a response.

Materials & experimental systems

n/a	Involved in the study	
☒	☐	Antibodies
☒	☐	Eukaryotic cell lines
☒	☐	Palaeontology and archaeology
☒	☐	Animals and other organisms
☐	☒	Human research participants
☐	☒	Clinical data
☒	☐	Dual use research of concern

Methods

n/a	Involved in the study	
☒	☐	ChIP-seq
☒	☐	Flow cytometry
☒	☐	MRI-based neuroimaging

Human research participants

Policy information about studies involving human research participants

Population characteristics	Enrolled patients were aged ≥18 years (≥20 years in Japan; male and female; age range 26–93) with previously untreated, EGFRm (Exon 19 deletion or L858R) locally advanced or metastatic NSCLC. Also as stated in the primary publication, patients with central nervous system metastases whose condition was neurologically stable were eligible; any previous definitive treatment or glucocorticoid therapy had to be completed at least 2 weeks before initiation of the trial treatment (Soria et al. NEJM 2018;387:113-125).
Recruitment	Eligible patients were recruited by investigators at study sites. Resistance analysis is limited to patients with detected ctDNA which is a known prognostic factor - we address this bias in the manuscript.
Ethics oversight	The study was approved by the institutional review board (IRB)/independent ethics committee (IEC) associated with each study centre. For the IRB/IEC names and addresses, please request. Study protocol available: Full study protocol available at https://astrazenecagrouptrials.pharmacm.com/ST/Submission/View?id=12356 (noted on page 14 of the manuscript). This study was performed in accordance with the ethical principles that have their origin in the

Declaration of Helsinki and that are consistent with International Conference on Harmonisation/Good Clinical Practice and applicable regulatory requirements and the AstraZeneca policy on bioethics. Informed consent was obtained from all patients prior to enrollment into the study.

Note that full information on the approval of the study protocol must also be provided in the manuscript.

Clinical data

Policy information about clinical studies

All manuscripts should comply with the ICMJE guidelines for publication of clinical research and a completed CONSORT checklist must be included with all submissions.

Clinical trial registration	NCT02296125
Study protocol	The full study protocol is available at https://astrazenecagrouptrials.pharmacm.com/ST/Submission/View?id=12356.
Data collection	Patients were recruited at 132 trial centers in Australia, Belgium, Brazil, Bulgaria, Canada, China, Czech Republic, France, Germany, Hungary, Israel, Italy, Japan, Republic of Korea, Malaysia, Philippines, Poland, Portugal, Romania, Russian Federation, Spain, Sweden, Switzerland, Taiwan, Thailand, Turkey, Ukraine, the United Kingdom, the United States of America, and Vietnam from August 2014 to September 2015 (Soria et al. NEJM 2018;387:113-125). Clinical data were analyzed with a cut-off date of June 12, 2017 and in these exploratory analyses of resistance mechanisms, plasma samples at progression or treatment discontinuation included in the paired analysis were collected up until March 2019.
Outcomes	As this was an exploratory analysis of resistance mechanisms, no pre-defined primary or secondary outcome measures were assessed in this analysis. For these exploratory analyses, serial plasma samples were collected at baseline, 2 weeks, 3 weeks, 6 weeks, 9 weeks, 12 weeks and every 6 weeks thereafter, as well as at disease progression and/or treatment discontinuation. Disease progression was assessed by the investigator, according to the Response Evaluation Criteria in Solid Tumors (RECIST) version 1.1, every 6 weeks for 18 months, then every 12 weeks until objective progressive disease. To identify acquired mechanisms of resistance, circulating tumor DNA (ctDNA) samples were evaluated from paired plasma samples from the same patient collected at baseline and following disease progression and/or treatment discontinuation using next-generation sequencing (Guardant Health, Guardant360 74 gene panel or GuardantOMNI 500 gene panel). All 74 genes on the Guardant360 panel were included in the GuardantOMNI 500 gene panel. All analyses from each patient (at baseline and following progression and/or treatment discontinuation) were reported only for genes included across panels used. Genomic alterations were identified using Guardant Health’s pipeline.<br>Primary mechanisms of resistance were also identified from tissue samples, collected at baseline in patients with and without detectable plasma EGFRm, using the FoundationOne CDx panel. Baseline tumor tissue samples were also used to analyze co-occurring mutations at baseline that would be associated with suboptimal response to osimertinib.<br>The duration of randomized treatment was defined as the time from randomization until end of EGFR-TKI treatment, and was determined for candidate resistance mechanisms in the osimertinib arm and presented as swimmer plots.
